# Biological Activities and Solubilization Methodologies of Naringin

**DOI:** 10.3390/foods12122327

**Published:** 2023-06-09

**Authors:** Hao Jiang, Mutang Zhang, Xiaoling Lin, Xiaoqing Zheng, Heming Qi, Junping Chen, Xiaofang Zeng, Weidong Bai, Gengsheng Xiao

**Affiliations:** 1 Guangdong Provincial Key Laboratory of Lingnan Specialty Food Science and Technology, Zhongkai University of Agriculture and Engineering, Guangzhou 510225, China; jianghao423@yeah.net (H.J.); luckxf_zeng@163.com (X.Z.); gshxiao@aliyun.com (G.X.); 2 Key Laboratory of Green Processing and Intelligent Manufacturing of Lingnan Specialty Food, Ministry of Agriculture, Zhongkai University of Agriculture and Engineering, Guangzhou 510225, China; 3 Academy of Contemporary Agricultural Engineering Innovations, Zhongkai University of Agriculture and Engineering, Guangzhou 510225, China; 4 College of Light Industry and Food Sciences, Zhongkai University of Agriculture and Engineering, Guangzhou 510225, China; mutang_731@163.com (M.Z.); m17665089186@163.com (X.L.); qshin1951@foxmail.com (X.Z.); 5 Science and Technology Research Center of China Customs, Beijing 100026, China; 6 Meizhou Feilong Fruit Co., Ltd., Meizhou 514600, China

**Keywords:** naringin, physiological activity, solubilization, solubility, bioavailability

## Abstract

Naringin (NG), a natural flavanone glycoside, possesses a multitude of pharmacological properties, encompassing anti-inflammatory, sedative, antioxidant, anticancer, anti-osteoporosis, and lipid-lowering functions, and serves as a facilitator for the absorption of other drugs. Despite these powerful qualities, NG’s limited solubility and bioavailability primarily undermine its therapeutic potential. Consequently, innovative solubilization methodologies have received considerable attention, propelling a surge of scholarly investigation in this arena. Among the most promising solutions is the enhancement of NG’s solubility and physiological activity without compromising its inherent active structure, therefore enabling the formulation of non-toxic and benign human body preparations. This article delivers a comprehensive overview of NG and its physiological activities, particularly emphasizing the impacts of structural modification, solid dispersions (SDs), inclusion compound, polymeric micelle, liposomes, and nanoparticles on NG solubilization. By synthesizing current research, this research elucidates the bioavailability of NG, broadens its clinical applicability, and paves the way for further exploration and expansion of its application spectrum.

## 1. Introduction

Naringin (NG) known as 4′,5,7-Trihydroxyflavanone 7-Rhamnoglucoside is a compound that falls under the classification of dihydroflavonoids. This complex compound comprises 4′,5,7-hydroxyflavone (saccharide ligand) conjoined with rhamnose-β-1,2-glucose [1]. The primary source of naringin (NG) is citrus fruit, although the concentration is significantly different across different species. For instance, *Citrus aurantium* subsp. (*C.*) *reticulata* boasts an NG content of 3383.6 μg/mL, while *C. bergamia*, on the other hand, contains a significantly lower quantity at 22.3 μg/mL. Notably, in *C. paradisi*, NG significantly surpasses other common citrus flavonoids, making it the dominant compound [2]. Meanwhile, NG is mainly found in the waste of fruit and vegetable products such as citrus peel. Yu Matsuo et al. obtained NG from *C. natsudaidai* peel waste extract with a yield of 23.8–27.0 mg/g dried material, which had a better odor than commercial citrus-flavor drinks [3]. Research on the biological activities of NG and the improvement of bioavailability is conducive to the comprehensive utilization of product waste and increases the added value of related products. 

The extraction methodologies of NG primarily encompass the organic solvent method [4], the aqueous solution method [5], alkali extraction, and the acid precipitation method [6]. However, traditional extraction methods present significant drawbacks, including prolonged processing time, biological toxicity, substantial solvent loss, and poor separation selectivity. These inefficiencies render these traditional methods unsuitable for large-scale production [7]. Therefore, to augment the extraction efficiency of polyphenols, physical- and chemical-assisted methods have been employed, including ultrasonic-assisted extraction and enzyme-assisted extraction. Employing ultrasonic waves and cellulase prompts rigorous vibration, leading to cell-wall disintegration, therefore exposing the encapsulated polyphenols [8]. This innovative approach has been proven effective by research conducted by Jolita Stabrauskiene et al., who reported that the combined use of ultrasound-assisted extraction with thermal hydrolysis as a solvent demonstrates superior efficiency in extracting flavonoids from citrus peels compared to the utilization of ultrasound-assisted extraction alone. The increase in flavonoid extraction efficiency was notable, rising from 17.45 ± 0.872 mg/g to 25.05 ± 1.25 mg/g [9]. The advancements in extraction techniques are significant as they open new avenues for applying NG. NG could be used as an additive in the food industry, and as a source of food sweeteners such as neohesperidin, dihydrochalcone, and naringin dihydrochalcone [10]. The continued exploration and refinement of these techniques will be essential for the optimization of the extraction and utilization of NG.

Meanwhile, NG has been identified as possessing a wide array of physiological effects, including anti-inflammatory, anti-oxidative, anticancer, anti-osteoporosis, and lipid-lowering properties. It has also been found to enhance the absorption of other drugs, therefore showcasing its potential as a pharmaceutical agent [11]. In the human body, the metabolism of NG involves a two-step process facilitated by the liver enzyme naringinase. The initial phase of this process involves the hydrolysis of NG by α-L-rhamnosidase, resulting in the formation of rhamnose and prunin. The subsequent phase entails hydrolysis by β-D-glucosidase, producing naringenin and glucose [12]. Interestingly, the 4th and 5th ligands of NG have been demonstrated to form a 1:1 complex with Cu (II). This complex enhances antioxidant, anti-inflammatory, and cytotoxic activities against tumor cells compared to free naringin, while maintaining cell viability [13]. 

NG is regarded as a new food raw material with high safety, and its safety has been identified in many respects. It has been reported that NG is practically non-toxic and there was no observed adverse effect level (NOAEL) in Sprague–Dawley rats following an oral acute toxicity study. Moreover, NG still shows a variety of physiological activities and non-toxic stability for fertility when administered by oral gavage with a 1250 mg/kg/day concentration for 13 consecutive weeks or 6 consecutive months [14]. However, the therapeutic potential of NG is significantly hindered by its low oral bioavailability, which is less than 5%. It has been found that after oral NG, almost no unaltered NG is found in the body. NG is metabolized by lactase-phlorizin hydrolase and intestinal microflora into naringenin. Meanwhile, most naringenin is in the form of naringenin glucuronide/sulfate. More specifically, NG in plasma reaches a maximum concentration (about 150 ng/mL) after 0.25 h of oral administration of 42 mg/kg NG in aged rats. Then, after oral administration of NG for 8 h, the concentration of naringenin obtained from naringin in the body reaches a maximum of approximately 2600 ng/mL [15]. After oral administration of 50 mg/kg NG for 6 h, the plasma concentration of NG in rats reaches the maximum of only 500 ng/mL [16]. This limitation has been attributed to NG’s low solubility and permeability, which curtails its applicability in the pharmaceutical and food industries. Consequently, enhancing the bioavailability of sparingly soluble NG has become a significant challenge, and a focal point of contemporary research [11]. The development of high-solubility NG preparation has been facilitated by advanced solubilization techniques, enhancing its absorption and physiological functions. Research has highlighted several solubilization approaches, including structural modification, solid dispersion (SD), encapsulation in amphiphilic molecular, emulsion, nanosuspension, liposome, and polymeric micelles technology [17]. To date, numerous in vivo and in vitro studies on NG have been conducted. Notably, the coadministration of a prodrug with NG resulted in a 1.35–1.69-fold increase in the relative bioavailability of paclitaxel compared to the prodrug alone. This increment was statistically significant (*p* < 0.05), improving the absolute bioavailability from 6.6% to 9.0% and 11.2% [18]. NG poses no obvious toxic harm to the human body after the use of solubilization approaches. Both the nanoparticle delivery system [19] and the newly developed α-glycoylated NG [20] overcame the insoluble limitation of NG, and its non-toxic effect has been used as an ideal carrier to effectively improve treatment efficiency and reduce side effects during treatment. The profound implications of these findings underscore the high application value and development potential of NG in various sectors, particularly within the food (as a food additive) and medica (in medical preparations) industries. This paper aims to provide a succinct overview of NG, outlining its physiological functions and the methodologies employed in its solubilization methods. Our objective is to foster NG’s continued development and diverse applications across multiple sectors.

## 2. The Biological Activities of Naringin

### 2.1. Anti-Inflammatory

NG exhibits potent anti-inflammatory properties, efficaciously mitigating acute inflammation instigated by pro-inflammatory factors. Overproduction of inflammatory mediators such as free radicals, cytokines, and chemokines, along with an escalated infiltration of inflammatory and immune cells, disrupt cellular and tissue functions and are associated with several acute and chronic diseases [21]. Specifically in macrophages, NG has been found to significantly suppress the production of inflammatory factors such as nitric oxide (NO), nitric oxide synthase (iNOS), cyclooxygenase-2 (COX-2), tumor necrosis factor-α (TNF-α), and interleukin 6 (IL-6). Moreover, NG also inhibits the activation of nuclear factor kappa-B (NF-κB) induced by lipopolysaccharide (LPS) in cells [22]. NF-κB serves as a pivotal mediator in cellular responses to external stimuli, often acting as an initial responder that potentially intensifies the expression of inflammatory factors [23]. In this context, NG’s role in inhibiting the activation of NF-κB has emerged as particularly significant, given its anti-inflammatory potential. Meanwhile, a study by Liu et al. demonstrated that NG alleviates the secretion of lung-tissue myeloperoxidase (MPO), iNOS activity, and TNF-α expression in a dose-dependent manner in LPS-induced inflammation mice. Concurrently, the degradation of the inhibitor of nuclear factor kappa-B-alpha (IĸB-α) and the translocation of protein NF-κB p65 are hindered, effectively inactivating NF-κB activation. These findings suggest that NG might exert substantial anti-inflammatory effects in lungs exposed to LPS, likely by inhibiting NF-κB activation [24]. Therefore, NG has been demonstrated to have significant potential as an effective anti-inflammatory agent.

### 2.2. Anti-Diabetes

Insulin resistance is a fundamental pathological feature of type 2 diabetes, often exacerbated by the impairment of insulin signaling. This impairment significantly aggravates diabetes symptoms by reducing insulin efficacy [25]. Research has demonstrated that NG ameliorates insulin resistance induced by a high-fat diet through the activation of the insulin pathway (phosphatidyl inositol 3 kinase (PI3K)/protein kinase B (PKB or AKT) and glucose transporters 4 (GLUT4) translocation. This activation induces GLUT4′s translocation to the plasma membrane), therefore facilitating a decrease in blood glucose levels [26]. In addition, NG enhances glucose uptake by promoting the translocation of GLUT2 in HepG2 cells under high glucose conditions. At the same time, it suppresses hepatic gluconeogenesis and promotes glycogen synthesis by activating the adenosine 5’-monophosphate (AMP)-activated protein kinase (AMPK) pathway. As a result, blood glucose is absorbed by the peripheral organs, effectively reducing blood glucose levels [27]. Therefore, NG could be used as a potential candidate for treating type 2 diabetes.

Moreover, NG has demonstrated potential for ameliorating diabetic nephropathy (DN), a typical complication of type 1 and type 2 diabetes. Zhang et al. found that NG suppressed the expression of NADPH oxidase 4 (NOX4) at both mRNA and protein levels through in vivo and in vitro model experiments of DN. The down-regulation of NOX4 notably reduces the expression level of Cleaved caspase3 in podocytes, resulting in significant suppression of apoptosis and reactive oxygen species levels. Meanwhile, it curtails the excessive accumulation of extracellular matrix (ECM) in mesangial and renal tubular cells, alleviating streptozotocin-induced oxidative stress injury and mitigating apoptosis and highly reactive oxygen species levels triggered by high glucose levels [28]. Therefore, NG has been demonstrated to have a therapeutic effect on DN complications, primarily through the mitigation of histiocytic symptoms associated with DN.

### 2.3. Hepatoprotective Activity

NG has been identified as a potential therapeutic agent in ameliorating symptoms of hepatic disease. Research conducted by Rossana Bugianesi et al. illustrated that NG is predominantly metabolized in the liver, and after oral ingestion of tomato paste rich in NG glycosides, it circulated in the body as a conjugated form [29]. This implied that NG sourced from plant-based foods is effectively absorbed by the body, enhancing its bioavailability. As a result, it can significantly contribute to preventing and treating liver disease in humans.

Research has shown that NG (25 mg/L) effectively reduces alcohol-induced lipid accumulation in the subcutaneous layers and liver of the zebrafish model. Meanwhile, hepatocyte steatosis was inhibited, and liver damage caused by fat deposition was improved [28]. In further research, fat deposition was improved by decreasing the formation and accumulation of reactive oxygen species (ROS) in the liver [30]. Meanwhile, NG has the potential to regulate oxidative pressure and inhibit hepatocyte injury. In the experiment, NG treatment at 5 mg/L for 48 h significantly reduced the alcohol-induced lipid droplet accumulation in the livers of exposed larvae with a dose-dependent tendency. More specifically, NG significantly reduced the oxidative stress gene expression of *cyp2y3* and *fabp10α*, which was induced by alcohol. Both oxidative stress genes were involved in the regulation of fatty acid metabolism and lipid uptake and transport. Furthermore, in zebrafish, the closest *cyp2e1* homolog was *cyp2y3*, which is 43% identical to the human protein. NG also reduced hepatocyte apoptosis of zebrafish larvae in experiments of terminal deoxynucleotidyl transferase-mediated dUTP-biotin nick end labeling (TUNEL) staining, and played a positive role in anti-apoptosis. Moreover, NG exhibits the physiological activity of anti-apoptosis. Some related factors that cause lipid metabolism disorder, endoplasmic reticulum stress, and DNA damage were downgraded due to the intake of NG, and the phenomenon of apoptosis was reduced [31]. 

In addition, NG has been demonstrated to have significant efficacy in mitigating nonalcoholic fatty liver disease (NAFLD). First, NG reduced hepatic steatosis in rats subjected to a high-fat diet. This hepatoprotective activity appeared to be partially mediated by the activation of the AMPK pathway. Upon the activation of the AMPK pathway, there was a consequent re-establishment of reduced antioxidant enzyme activities, comprising superoxide dismutase (SOD), catalase (CAT), glutathione peroxidase (GPx), and glutathione S-transferase (GST), as well as prevented inflammation [32]. The increase in antioxidant enzyme activities enhances liver detoxification and protects the liver from free-radical damage [33]. Meanwhile, NG has been shown to have significant potential in ameliorating the symptoms of cadmium-induced hepatotoxicity (at a concentration of 50 mg/kg NG) and nickel-induced hepatotoxicity (at 80 mg/kg NG) (Table 1). These were demonstrated by a notable decrease in lipid peroxidation and a substantial increase in the activity of antioxidant enzymes in rats [34]. Moreover, naringenin (50 mg/kg, an aglycone of NG) was shown to have a remarkably restorative effect on liver function abnormalities induced by dimethylnitrosamine (DMN) in mice [35].

In summary, the demonstrated hepatoprotective activities of NG substantiate its potential as a promising therapeutic candidate for liver diseases, highlighting its value for further clinical research.

### 2.4. Neuroprotective Activity

NG has been demonstrated to have tremendous potential for the prevention and therapeutic intervention of neurodegenerative diseases such as Parkinson’s disease, epilepsy, Huntington’s disease, and Alzheimer’s disease (AD) (Table 1). Investigations have revealed that NG has a protective effect on viable neurons affected by 3-nitropropionic (3-NP), a succinate dehydrogenase inhibitor known to induce neuronal injury and death in rats. This protective effect has been demonstrated by the reduction of radical species such as hydroxyl radical (29.36%), hydroperoxide radical (36.32%), and nitrite radical (45.62%) in 3-NP-treated rats. Moreover, NG ameliorated the tissue damage caused by 3-NP exposure by enhancing the GSH/GSSG ratio value by 74.17%. Additionally, NG significantly increased mRNA expressions of HO-1, NQO-1, GST-P1, and c-GCL by 60.78%, 72.5%, 64.71%, and 55.79%, respectively, in the treated rats compared with the 3-NP-induced group. Impressively, these expressions exceeded 75% compared to the control group of rats [36]. These increases were significant since HO-1, NQO-1, GST-P1, and c-GCL were induced by the nuclear erythroid 2-related factor 2 (Nrf2) [37,38]. Consequently, the pronounced neuroprotective effect of NG can be attributed to its potent antioxidant activity, mediated through the Nrf2 signaling cascade. NG has been seen to protect neurons by stimulating the production of neurotrophic factors and activating the Nrf2 signal transduction pathway [39]. In the case of AD patients, there is typically an overactivation of pro-inflammatory M1-type cells in the brain tissue, leading to excessive secretion of pro-inflammatory factors and cytotoxic substances [40]. NG has been shown to mitigate cognitive impairment in AD patients by regulating the balance of pro-inflammatory cells (M1 type) and anti-inflammatory cells (M2 type). Furthermore, NG improves abnormal behavioral features by enhancing the phagocytosis and clearance of Aβ-oligomer, therefore offsetting neurotoxicity and neuroinflammation [41]. This finding has established a theoretical framework for treating AD and presents a novel therapeutic approach. In short, NG offers promise as a prospective medication for treating and preventing neurodegenerative disorders.

### 2.5. The Drug Absorption Enhancer

NG and its derivatives play roles as solubilizers, boosting the absorption of other drugs, and elevating their bioavailability, as demonstrated in drugs such as paclitaxel, diltiazem, candesartan, and pranlukast. Paclitaxel, an anticancer drug, is characterized by poor solubility and challenging absorption into the body, predominantly due to the exocytosis of p-glycoprotein (p-gp) at the top of the intestinal epithelial cells [42]. Owing to these complexities, paclitaxel is mainly administered through intravenous injection, which, unfortunately, is prone to induce allergic reactions, therefore limiting its clinical application. However, studies have reported an elevated plasma concentration of paclitaxel when co-administered with NG’s prodrug 7-mPEG 5000-succinyloxymethyloxycarbonyl-paclitaxel, compared to when paclitaxel was administered alone [18]. This phenomenon suggests that NG effectively improves the bioavailability of paclitaxel, presenting a promising avenue for the development of oral paclitaxel medicines. Similarly, NG also enhances the bioavailability of diltiazem, a calcium-channel antagonist. It has been shown that metabolic enzymes and p-glycoprotein impede the absorption of diltiazem [43]. However, upon administration of NG (5 or 15 mg/kg), both the area under the plasma concentration–time curve (AUC) and the peak concentration (C_max_) have been observed to double, indicating significant changes in pharmacokinetics and a marked improvement in bioavailability. In addition, Surampalli et al. explored the effect of NG on the absorption of candesartan (an antihypertensive drug) within the intestinal tract of rats using a single-channel perfusion model (Table 1). Their results illustrated that the lyophilized solid dispersion of NG significantly increases the maximum concentration (C_max_) of candesartan and shortens the time (t_max_) to reach C_max_ compared to when the drug was administered alone. This outcome was attributed to low concentrations of NG substantially inhibiting the function of p-glycoprotein [44]. Moreover, α-glycosylated NG significantly increased the apparent solubility of pranlukast hemihydrate (PLH, a drug used to treat bronchial asthma) in distilled water at 37 °C, as per the dissolution test. The apparent solubility of PLH increased from 0.17 ± 0.01 μg/mL in the control group to 14.54 ± 1.32 μg/mL, and this increase was proportional to the concentration of α-glycosylated NG [20]. Concurrently, the AUC of the physical mixture of PLH with α-glycosylated NG was 2.2 times greater than that of the PLH treatment alone in a rat model, indicating that α-glycosylated NG improved the oral absorption of PLH [20]. In summary, NG has emerged as a potential active ingredient capable of improving the bioavailability of drugs with poor solubility, reinforcing its pivotal role in drug absorption enhancement.

These characteristics of NG, which greatly limit its application in practical production, mainly include low solubility, low permeability, short half-life, high plasma concentration fluctuation, bitter taste, and toxicity at high concentrations (≥200 μg/mL) [11]. Therefore, the solubilization of NG and the improvement of its bioavailability have become research hotspots. The solubilization methods of NG are summarized in the following table, including structural modification, solid dispersion, liposomes, preparation of nanoparticles, and amphiphilic molecular encapsulation, to provide a reference for solving NG’s poor-solubility problem.

**Table 1:** The physiological activities of naringin.

Physiological Activities	Constituent	Dose	Animal Model	Potential Mechanisms	References
Anti-inflammatory	NG	100 mg/kg	HFD-induced obesity mice	**Decrease:** Mac-2, MCP-1, JNK phosphorylation	[45]
		36.8 mg/kg	CS-induced chronic bronchitis in guinea pigs	**Increase:** Activities of SOD and LXA4 **Decrease:** IL-8, LTB4, TNF-α, BALF, and myeloperoxidase activity	[46]
		60 mg/kg	LPS-induced endotoxin shock in mice	**Decrease:** NO, TNF-α, IL-6, iNOS, COX-2 and transcriptional activity of NF-κB	[47]
		3 mg	LPS/D-galactosamine-induced liver injury mice	**Decrease:** AST, ALT, CK, TNF-α	[48]
Anti-diabetes	NG	30 mg/kg	STZ-induced diabetic mice	**Increase:** Activity of hexokinase **Decrease:** Activities of glucose-6-phosphatase and fructose-1,6-bisphosphatase in the liver and kidney	[49]
		200 mg/kg	C57BL/KsJ-db/db mice (Diabetic mouse model)	**Increase:** Hepatic glucokinase activity and glycogen concentration **Decrease:** Activity of hepatic G6-P and phosphoenolpyruvate carboxykinase	[50]
	naringenin	50 mg/kg	STZ-nicotinamide–induced diabetes mice	**Increase:** Serum insulin concentrations **Decrease:** Activities of ALT, AST, ALP, and LDH in serum, Concentrations of fasting blood glucose, Glycosylated hemoglobin	[51]
	NG	50 mg/kg	HFD/STZ-nicotinamide–induced diabetes mice	**Increase:** G6Pase, Glycogen phosphorylase, FBPase, Insulin release **Decrease:** MDA, NO, TNF-α, IL-2	[52]
Hepatoprotective activity	NG	80 mg/kg (Nickel) and 50 mg/kg (Cadmium)	Nickel and Cadmium-induced hepatotoxicity in mice	**Increase:** SOD, CAT, GPx, GST, GST, GSH, vitamin C, and vitamin E **Decrease:** AST, ALT, ALP, LDH, GGT, TB, The liver nickel concentration, Lipid peroxidation indices, and protein carbonyl contents	[34,53]
	naringenin	50 mg/kg	DMN-induced liver injury mice	**Increase:** Body weight, Serum albumin, and total protein levels **Decrease:** ALT, AST, ALP, and bilirubin levels, MDA, Hepatic stellate cell activation	[35]
	NG	20 mg/kg	APAP induced in male Wistar mice	**Increase:** Albumin, IL-4, GSH, SOD, GST, GPx, Bcl-2 **Decrease:** AST, ALT, ALP, LDH, GGT bilirubin, lipid, TNF-α, lipid peroxidation p53, Bax, CASP-3	[54]
	naringenin	25 mg/L	2% ethanol-induced larvae of zebrafish	**Increase:** Cyp2y3 and Fabp10α, Histological injury severity, Apoptotic cell death, and SOD radical levels	[55]
	NG	100 mg/kg	5-fluorouracil induced liver and kidney toxicity in mice	**Increase:** GSH, SOD **Decrease:** ALT, AST, ALP, MDA, IL-1α, TNF-α, IL-6	[56]
Neuroprotective Activity	NG	80 mg/kg	3-NP-induced neurodegenerative disease in mice	**Increase:** Nuclear translocation of Nrf2, Induce phase II genes such as HO-1, NQO-1, GST-P1 and γ-GCL expression **Decrease:** TNF-α, COX-2, and iNOS mRNA expression	[36]
		80 mg/kg	KA-induced neurodegenerative disease in mice	**Increase:** Protected hippocampal CA1 neurons, the expression of LC3 **Decrease:** TNF-α, Occurrence of SRS	[57]
		100 mg/kg	Aβ-induced AD mice	**Increase:** CaMKII activity, Phosphorylation of AMPA, Improved long-term learning and memory ability **Decrease:** GSK-3β activity	[58]
		200 mg/kg	ICV-STZ-induced AD mice	**Increase:** CAT, SOD, GSH, Mitochondrial complex (I, II, and IV) **Decrease:** Cholinesterase activity, MDA, nitrate level, TNF-α, IL-1β	[59]
The drug Absorption Enhancer	PLH/Naringin-G	PLH 40 mg/kg/NG 80 mg/kg	Male Sprague–Dawley mice	**Increase:** PLH solubility and absorption	[20]
	NG	15 mg/kg	in-situ rat models	**Increase:** Candesartan absorption, AUC value, and C_max_value **Decrease:** t_max_value, the release of protein and ALP	[44]
	Preparation of GGTN composite with NG	10 mg/mL	Rabbit skull defect model	**Increase:** Bone regeneration, bone conduction activity, new bone growth, wound healing	[60]

Abbreviations: HFD, High-fat diet; MCP-1, monocyte chemoattractant protein-1; JNK, C-Jun N-terminal kinases; CS, Chronic cigarette smoke; SOD, superoxide dismutase; LXA4, the content of lipoxin A4; IL-8, Interleukin-8; LTB4, leukotriene B4; TNF-α, Tumor necrosis factor α; BALF, bronchoalveolar lavage fluid; MPO, myeloperoxidase activity; LPS, lipopolysaccharide; NO, Nitric oxide;IL-6,Interleukin-6; iNOS, inducible nitric oxide synthase; COX-2,cyclooxygenase; NF-kB, kappa-light-chain-enhancer of activated B cells; ALT, alanine transaminase; AST, aspertate transaminase; CK, creatine kinase; STZ, streptozotocin; G6-P, glucose 6-phosphate; G6Pase, Glucose-6-phosphatase; FBPase, fructose-1, 6 bisphosphatase; MDA, malondialdehyde; PLH, pranlukast hemihydrate; IL-2,Interleukin-2; LDH, lactate dehydrogenase; GGT, γ-glutamyl transferase; TB, serum total bilirubin; CAT, catalase; GPx, glutathione peroxidase; GST, glutathione S-transferase; GSH, glutathione; ALP, alkaline phosphatase; DMN, dimethylnitrosamine; APAP, N-Acetyl-p-aminophenol; IL-4, Interleukin-4; Bcl-2, lymphoma 2; Bax, Bcl-2 associated X; CASP-3, cysteine aspartate-specific protease-3; cyp2y3, cytochrome P450 family 2 subfamily Y polypeptide 3; Fabps, Fatty acid-binding proteins; IL-1α, interleukin-1α; 3-NP, 3-nitropropionic acid; Nrf2, Nuclear factor-erythroid 2-related factor-2; HO-1,heme oxygenase-1; NAD(P)H, quinone oxidoreductase-1; NQO-1, NAD(P)H: quinone oxidoreductase-1; GST-P1,glutathione S-transferase P1; γGCl, γ-glutamylcysteine ligase; KA, Kainic acid; LC3, microtubule-associated protein light chain 3; SRS, spontaneous recurrent seizures; Aβ, Amyloid-β; AD, Alzheimer’s disease; CaMKII, calcium/calmodulin-dependent protein kinase II; AMPA, α-amino-3-hydroxy-5-methyl-4-isoxazolepropionic; GSK-3β, Glycogen synthase kinase-3β; PLH, pranlukast hemihydrate.

## 3. The Methods of Solubilization

### 3.1. Structure Modification

Structural modification is a strategy that entails altering functional groups while preserving the foundational structure of a compound, therefore not compromising its essential properties [61]. This process achieves beneficial outcomes such as mitigating adverse effects, modifying targets, and enhancing bioavailability by adding, subtracting, and replacing specific groups [62]. At present, the primary modes of structural modification in flavonoids include glycosylation, acylation, sulfonation, and methylation, which endow flavonoids with exceptional characteristics [63]. In the context of NG, selective modification has proven instrumental in enhancing its physical and chemical properties, such as water and fat solubility. Moreover, this selective modification also positively influences the physiological attributes of NG, including its intestinal absorption rate and antioxidant efficacy. Consequently, structural modification improves the solubilization and stability of the modified target, preserving the integrity of the original active structure and the safety profile of NG.

#### 3.1.1. Acylation

**Figure 1:**
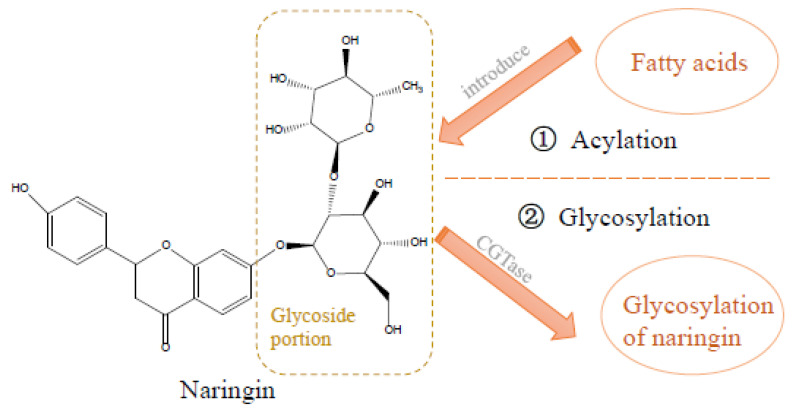
The main methods for structural modification of naringin are acylation and glycosylation, which show that naringin possesses excellent properties. Lipid solubility is focused on the modification of the acylation structure. ① The acylation reaction occurs in the glycoside region. Chemical and enzymatic methods have been used to introduce fatty acids into the glycoside portion of naringin to improve its lipid solubility. ② In addition, relating to water solubility, naringin can be glycosylated with cyclodextrin glucanotransferase (CGTase) in an alkaline environment, at which stage the solubility of the glycosylated naringin is significantly increased. Thus, the selective modification of naringin improves its water and fat solubility. Based on data from [67] Almeida, et al. (2012), [68] Kometani, et al. (1996), and [69] Lee, et al. (1999).

Focusing on lipid solubility enhancement, alterations in acylation structures have been explored as viable strategies. More specifically, both chemical and enzymatic methods have been employed to introduce various fatty acids, including saturated fatty acid, unsaturated fatty acid, substituted fatty acid, dicarboxylic acid, and aromatic acid, into the glycoside portion of NG to improve its lipid solubility [64] (Figure 1). However, these methodologies present distinct advantages and drawbacks. Chemical acylation processes have often been criticized for their complex steps, significant pollutant output, and high energy consumption. Conversely, enzymatic methods exhibit high selectivity, yielding a diverse range of products [65]. Among them, the inability to precisely determine the acylation position represents a challenge within the diversity of the enzymatic products [66]. Nevertheless, the specific acylation of the glycosides within NG can be accomplished through catalysis using an immobilized enzyme in a non-aqueous solvent. Empirical evidence suggests that NG could be successfully acylated under certain conditions to achieve superior lipid solubility. One study showed that when the ratio of acylating agent to NG was 3:1 (acetone was used as solvent), immobilized candida antarctica lipase catalyzed the reaction between naringin and unsaturated castor oil acid at 50 °C for 120 h. This reaction yielded acylation products with significantly enhanced lipid solubility, primarily comprising naringin 6′-ricinoleate. Notably, the acylation reaction occurred in the glycoside region, therefore retaining the flavonoid structure was responsible for its physiological activities such as antioxidant and anti-inflammatory [67]. The resultant acylated NG was obtained through process optimization and demonstrated heightened lipid solubility. This enhanced lipid solubility significantly improved NG’s ability to permeate cell membranes, consequently improving its overall bioavailability.

#### 3.1.2. Glycosylation

The solubility enhancement of flavonoids via glycosylation represents a crucial area of research. This approach, which involves altering the type and content of carbohydrates (glucose, galactose, cellobiose, maltose, and rhamnose) has been found to increase the hydrophilicity of these compounds, therefore enhancing their solubility in water [64]. Kometani et al. accomplished the glycosylation of NG using cyclodextrin glucanotransferase (CGTase) under alkaline conditions. The solubility of the resultant product increased to a level 1000 times higher than that of NG aglycone, while the bitterness remained relatively unaltered [68]. Meanwhile, another significant breakthrough was observed when 6^G^-α-maltosyl naringin, a product of glycosylation of NG by maltogenic amylase from *Bacillus stearothermophilus*, demonstrated enhanced water solubility 250 times greater than that of NG. Furthermore, its bitterness was significantly reduced to merely a tenth of NG’s, due to the variation in solubility of its enzymatic hydrolysis product from that of CGTase [69]. These findings underscore the significant influence of enzyme choice on the glycosylation position, therefore affecting the physical properties of NG and the group incorporated into the product after enzymatic hydrolysis. Furthermore, the molecular weight of NG is also affected by the number of glycosides after glycosylation. It has been reported that the greater the number of glycosides after glycosylation of anthocyanin, the higher the molecular weight and lower the bioavailability. Meanwhile, there is also a significant difference in bioavailability when carrying the same number of but different glycosides [70]. Therefore, the obtaining of an NG complex with high solubility and bioavailability would be expected if fewer glycosides that were more easily absorbed by the human gastrointestinal tract were introduced during glycosylation. 

It has been extensively studied that acylation and glycosylation have been used to improve the solubility of flavonoids with poor water solubility. Most flavonoids are rich in physiological activity. If the bioavailability of flavonoids such as NG can be increased along with solubility, the medicinal value of flavonoids will be greatly enhanced.

### 3.2. Solid Dispersion 

**Figure 2:**
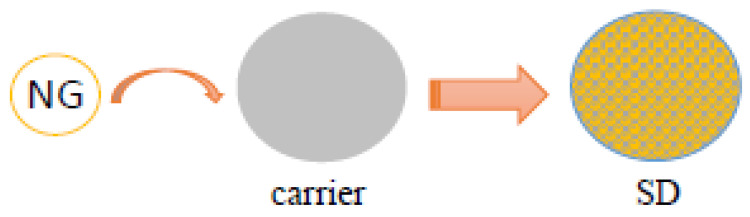
Naringin dispersed in a solid carrier (SD) existing in the amorphous form. Solubility and dissolution rates of naringin increase with the surface area for dissolution and increase by reducing crystallinity to an amorphous state. Therefore, the dissolution rate and oral bioavailability of naringin are greatly improved in NG-PEG6000 SD. Based on data from [74] Sinha, et al. (2010), [75] Gigliobianco, et al. (2018), [76] Wang J, et al. (2018), and [77] Kanaze, et al. (2006).

Solid dispersion (SD) is a unique method wherein the target compound can exist in a molecular, amorphous, or microcrystalline state, dispersed within a solid carrier [71] (Figure 2). This methodology enhances the solubility and dissolution rates of the target compound by increasing the surface area for dissolution, reducing crystallinity to an almost amorphous state, and increasing drug wettability [72]. However, employing a single material as a carrier may present certain limitations. Therefore, a blend of two or more carrier materials, each possessing distinct hydrophilic, hydrophobic, and enteric properties, is typically employed to maximize the solubility of the target compound [73].

Despite the potential of solid dispersion (SD) in the solubilization of NG, several challenges have been encountered during its preparation process, which constrains its broader application. Traditional preparation methodologies primarily incorporate the melt and solvent methods. Notably, the melt method necessitates high-temperature conditions, while excessively high temperatures can prompt the decomposition of target compounds and the carrier, impacting the thermally unstable carrier and compound [74]. Conversely, the solvent method encounters compatibility issues due to the contrasting hydrophilic and hydrophobic characteristics of the solvent-based carriers and the target compounds [78]. Furthermore, the properties of SDs have been affected by various parameters such as melting temperature, cooling rate, solvent nature, compound-to-solvent ratio, carrier-to-solvent ratio, drying method, and drying rate [79]. The dissolution rate of the compound is also affected by the particle size and compound-to-carrier ratio [80]. In addition, in terms of structure, although amorphous compounds display enhanced water solubility compared to crystalline drugs, their poor stability poses challenges. Issues have been raised as a result of the tendency of amorphous compounds to revert to a crystalline phase and enter supersaturation, as well as their susceptibility to crystallization during aging [81]. Moreover, large-scale heating and solvent evaporation requirements pose challenges such as inefficient space utilization [82]. Therefore, innovative technologies (supercritical fluid technology, spray-drying technology, hot melt extrusion technique, microwave quenching technology, and micro-environment pH control technique) have been progressively employed in the preparation of SDs [83]. These new technologies have many advantages compared with traditional SD processes. For example, the hot melt extrusion technique has the advantage of flexibility in process design, short process residence times, and is environmentally safe, as no organic solvents are employed during the process [84]. In addition, supercritical fluid technology, spray-drying technology, and micro-environment pH control technique did not require the high temperatures and more chemical solvents that are used in traditional processes. These technologies require a relatively low stability of processed samples and are environmentally safe [85]. At times, a combination of these techniques has been utilized to balance the stability and bioavailability of the compound [75]. Wang et al. employed polyethylene glycol 6000 (PEG6000), pluronic F68, and polyvinyl pyrrolidone K30 (PVP K30) as carriers while controlling the compound-loading ratio to prepare NG-SD. In a 1:1 to 1:5 carrier-to-compound ratio, the solubility of naringin noticeably increased, especially with PVP K30. At a 1:5 ratio, the solubility of NG in PVP K30 was almost three times that of the other two carriers [76]. However, an in vitro dissolution test revealed that SD significantly improved the dissolution rate of NG compared to its physical mixture. Specifically, NG dissolution in NG-PEG6000 SD exceeded 90% within 12 min, as opposed to approximately 70 min for NG-PVP K30 SD [76]. This enhanced dissolution rate and oral bioavailability of naringin have been further validated using a differential scanning calorimeter (DSC) and powder X-ray diffraction (PXRD) analysis, which confirmed the predominantly amorphous nature of naringin in NG-PEG6000 SD [76]. In the study by Kanaze et al., PEG and PVP were used as carriers to prepare SDs of flavanone aglycones (naringenin and hesperetin). The flavanone aglycones were crystalline in PEG-SD but existed in an amorphous, nano-disperse state in PVP-SD [77]. This phenomenon indicates that PVP demonstrates higher compatibility than flavanone aglycones. Moreover, the compound release rate of PVP-SD was higher than that of PEG-SD in the majority of carrier compound formulations [77]. Therefore, it is essential to evaluate the compatibility of NG and the carrier before choosing the carrier material for SD preparation. Additionally, the properties of compound solubility, in vitro dissolution, and stability of the prepared SD were comprehensively examined. Overall, PEG6000 shows potential application value in the preparation of NG-SD.

In the studies of pharmacokinetics, NG and other flavonoids with low bioavailability find it difficult to enter the target in effective doses. However, in rather complex and suitable solvents, the toxicity of the solvent to organisms is an unavoidable problem. Many studies have reported progress in improving the bioavailability of flavonoids by SD. Therefore, NG is expected to improve the dispersion property of NG through SD, thus improving the stability and post-delivery performance of NG in vitro and in vivo.

### 3.3. Inclusion Compound and Polymeric Micelle

#### 3.3.1. Cyclodextrin Inclusion Compound

**Figure 3:**
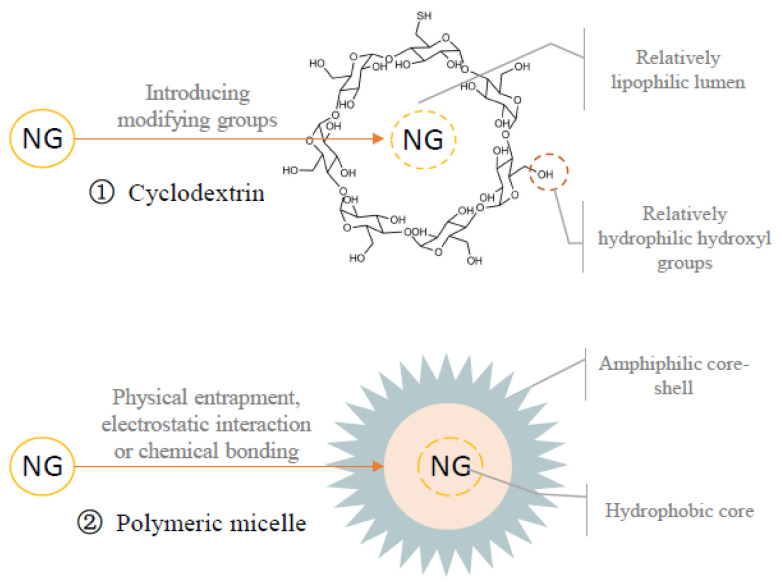
The amphiphilic molecules are embedded with naringin and aggregated into micelles, vesicles, and nanoparticles, increasing the solubility of naringin in water. ① In terms of CD structure, the external hydroxyl groups are relatively hydrophilic, ensuring solubility in aqueous systems. ② Moreover, amphiphilic polymers spontaneously are assembled into core–shell structures in selective solvents. Naringin is combined with the hydrophobic part, which is located inside amphiphilic polymers to form polymer micelles. By interacting with amphiphilic molecules, the solubility in water of naringin increased markedly. Based on data from [94] Shulman, et al. (2011), [95] Stasiłowicz-Krzemień, et al. (2022), [96] Abasian, et al. (2021), [97] Qu, et al. (2018), [98] Chen, et al. (2021), [87] Xiang, et al. (2021) and [99] Fan, et al. (2020).

Cyclodextrin (CD) is a crystal structure of 6–12 D-glucopyranose units linked by α-1,4-glucosidic bonds [86]. In terms of CD structure, the external hydroxyl groups are relatively hydrophilic, ensuring solubility in aqueous systems, suggesting that CD is typical the amphiphilic molecules [87,88]. The commonly employed variants for application are α-CDs, β-CDs, and γ-CDs, consisting of 6, 7, and 8 glucose units, respectively [89]. Figure 3 illustrates the spatial configuration of CDs: a cylindrical three-dimensional toroidal structure with a subtly conical hollow shape. In addition, the lumen of the CDs is relatively lipophilic and can encapsulate lipophilic substances, therefore modifying the physical and chemical attributes of the embedded compounds [90]. However, certain limitations of CD, such as cavity size and the confined limitation of its hydrophobic region, along with catalytic activity, necessitates a research shift towards CD derivatives with modifying groups (amination, etherification, or esterification of primary and secondary hydroxyl groups in CD) [91]. 

Furthermore, the combination of two or more different CDs, such as a β-CD and γ-CD blend, demonstrates superior solubilization effects compared to a single CD, owing to their impressive additive and cooperative properties [92]. For instance, Cui et al. synthesized an inclusion compound of naringin/β-cyclodextrin (the molar ratio was 1:1). This compound displayed water solubility at 37 ± 0.1 °C that was 15 times higher than that of free NG, as verified by differential scanning calorimetry (DSC) thermograms. This suggests that β-CD inclusion significantly increases the solubility of NG [93]. In addition, 2-hydroxypropyl-β-cyclodextrin (HP-β-CD) exhibits characteristics such as low toxicity, valuable thermal stability, and enhanced water solubility compared to β-CD. At the same time, HP-β-CD does not cause irritation symptoms or adverse reactions with hemolytic biofilms, and is used as a preparation component of various compounds [94]. A study showed that HP-β-CD increased the solubility of NG by more than 400 times, and accelerated the transport rate in an intestinal epithelial cell model by 11 times [94]. Stasiłowicz-Krzemień et al. prepared physical mixtures of NG with β-CD, HP-β-CD and hydroxy propyl methyl cellulose (HPMC) using the coprecipitation method, with NaHCO_3_ added as the second excipient in the solubilizer. It was observed that a hydrogen bond was formed between the phenolic hydroxyl group of the ring of NG and the hydroxyl group of HP-β-CD [95]. Furthermore, infrared spectroscopy analysis suggests that the aromatic ring of NG is contained within the interior of HP-β-CD, forming an inclusion complex between HP-β-CD and NG [95]. Meanwhile, some hydrogen bonds are dissociated in the alkaline micro-environment by NaHCO_3_, resulting in higher solubility of ternary SDs than binary SDs. Therefore, NG-HP-β-CD exhibited the highest solubility rate (458.3-fold increase in solubility) [95]. Overall, the NG-HP-β-CD inclusion complex exhibits significant potential for further development.

#### 3.3.2. Polymeric Micelle

Amphiphilic polymers, characterized by their spontaneous assembly into core–shell structures within selective solvents, have a combination of hydrophilic and hydrophobic interactions, intermolecular hydrogen bonds, and van der Waals forces [100]. This arrangement enables hydrophobic substances such as NG to interact with the hydrophobic part housed within these amphiphilic polymers, leveraging physical entrapment, electrostatic interaction, or chemical bonding to form polymer micelles (Figure 3). These micelles occur in a variety of forms, including grafting polymer micelles, block polymer micelles, and polyelectrolyte micelles, with each type offering its unique functional advantages [101]. Furthermore, polymeric micelles have been acknowledged for their distinct properties, such as easy preparation, low toxicity, favorable biocompatibility, and the accessible modification of surface groups [102]. Beyond their solubilization potential, they have been recognized for their controlled sustained release capability and targeting [96,97].

Esterified starch, an exemplar of amphiphilic polymers, possesses a hydrophilic backbone and hydrophobic branches. This polymer is synthesized by reacting hydrophilic starch molecules with hydrophobic octenyl succinic anhydride (OSA) groups [98]. In aqueous media, esterified starch forms monodisperse spherical ice crystals, and the resulting micellar structure is easily influenced by factors such as molecular weight and the presence of other organic compounds [103]. For instance, among five OSA-esterified waxy corn starches (OSAS) with different molecular weights, the medium molecular weight OSAS (M-OSAS) demonstrates the highest NG content and encapsulation efficiency. Meanwhile, in the complex NG-M-OSAS (50 mmol/L), the solubility of NG increases by 848.83 times [87]. In addition, (methoxy poly (ethylene glycol)-poly (ε-caprolactone) (MPEG-b-PCL) is an amphiphilic deblock copolymer known for its capability to self-assemble into core–shell micelles in an aqueous solution. Herein, poly (ε-caprolactone) (PCL) forms the inner core, enabling the efficient loading of hydrophobic substances [99]. Several studies have demonstrated the successful preparation of NG-loaded polymeric micelles (NGMs) via the thin-film rehydration method, then further freeze-dried to yield nanoparticle powder (23.95 ± 0.51 nm). This conjugate compound had the characteristics of high compound-loading, favorable encapsulation efficiency, strong solubility, and minimal human irritation and toxicity. Notably, integrating these lyophilized NGMs into orally disintegrating tablets significantly increases the solubility of NG [99]. Therefore, the utilization of polymer micelles for enhancing the solubility of NG is an area warranting further exploration. 

These natural or synthetic amphiphilic molecules possess both hydrophilic and hydrophobic groups. Their active constituents, whether at the terminal ends, trunk, branched chains, or within a spherical structure with a hydrophobic interior and hydrophilic exterior, can effectively encapsulate hydrophobic active substances such as NG [104]. Meanwhile, the amphiphilic molecules form aggregated structures such as micelles, vesicles, and nanoparticles upon encapsulating NG. This aggregation enhances the water solubility of NG [76]. Various natural and modified substances, including carbohydrates, proteins, phospholipids, phenols, and synthetic compounds, can be used as amphiphilic molecules [105]. The method of embedding NG into amphiphilic inclusion complexes (CD inclusion complexes and polymer micelles) effectively improved the solubility of NG. In addition, CD inclusion complexes and polymer micelles enhance the transport rate of NG in vivo within a safe range of toxicity. Therefore, in future research, a polymeric micelle approach should be developed to improve the sustained and targeted release of NG.

### 3.4. Liposome and Nanoparticles

#### 3.4.1. Naringin Liposome

**Figure 4:**
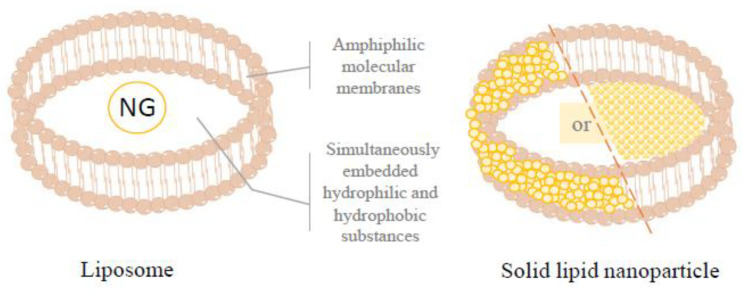
Liposomes are microvesicles composed of single or multi-layer amphiphilic molecular membranes and similar to cellular structures, which tend to simultaneously embed hydrophilic and hydrophobic substances. Therefore, the solubility of naringin liposome is higher than naringin. Furthermore, solid lipid nanoparticles (SLN) combine the excellent characteristics of liposomes and nanoparticles. The formulation of the nano in naringin liposomes enhances the bioavailability and specific delivery of naringin. Based on data from [109] Villa, et al. (2015), [116] Ji, et al. (2016) and [117] Elkhoury, et al. (2020), [119] Bhia, et al. (2021), and [120] Yang, et al. (2020).

Liposomes are microvesicles composed of single or multi-layer amphiphilic molecular membranes, similar to cellular structures, making them efficient carriers for both hydrophilic and hydrophobic substances [106] (Figure 4). For instance, GEO-loaded phytosomes are used as potential natural preservatives exhibiting antimicrobial activity in yogurt products, and as a potential food additive for enriching food products with water-insoluble nutraceuticals. [105]. A similar application can be noted for steppogenin, a naturally occurring flavanone with robust tyrosinase inhibitory activity, predominantly found in Moraceae plants. Like NG, steppogenin’s water solubility is relatively low. However, Tao et al. successfully improved steppogenin’s solubility and stability in aqueous systems using O/W emulsions prepared from Saponin-loaded steppogenin. The study revealed a remarkable enhancement in steppogenin’s solubility, up to 3000 times higher than its water solubility. This innovative solubilization method might significantly influence future research and industrial development in the food-processing industry, particularly concerning the application of NG [107]. 

Phospholipids and surfactants are amphiphilic molecules that can self-assemble through various non-covalent chemical interactions. This unique property enables them to release their encapsulated structures at target sites in a controlled, sustained manner, therefore enhancing the stability and bioavailability of the encapsulated compounds [108]. First, traditional liposomes are established as closed spherical bilayers formed by phospholipids and cholesterol [109]. This configuration improves the solubility, stability, bioavailability, and other physical properties of the entrapment. However, their susceptibility to external factors, such as light, temperature, and oxidation, gives rise to instability, manifesting as liposome cracking, fusion, and aggregation [110]. Subsequent research has focused on the creation of novel liposomes by hybridizing existing liposomes and adjusting the materials and methods employed in their preparation. This has led to the development of modified liposomes, membrane fusion liposomes, and deformed liposomes [111]. 

Recent studies have demonstrated that the attachment of polymers, antibodies, or phospholipid derivatives to the surface of liposomes effectively improves liposome properties and their storage stability [112,113,114]. Frequently used liposome modifiers include chitosan, alginate, polyethylene glycol, pectin, and silica. In addition, the stability of liposomes is considerably improved by forming films through polyelectrolyte deposition on their surfaces [115]. By extension, the NG liposomes prepared using the double-emulsion method and the emulsification–low-temperature-curing method have been recognized for their high encapsulation efficiency, simplicity of operation, and remarkable stability [116]. Furthermore, in a study by Elkhoury et al., NG nanoliposomes were prepared from salmon lecithin, known for its richness in unsaturated fatty acids and bone-protective effects. The NG nanoliposomes displayed commendable attributes such as high encapsulation efficiency (99.7 ± 0.07%), nanoscale size (114 nm), and slow NG release [117]. Meanwhile, toxicological tests indicated that NG nanoliposomes at concentrations of 50 μg/mL and 100 μg/mL (equivalent to NG in human adipose stem cells at concentrations of 25 μg/mL and 50 μg/mL) did not exhibit significant cytotoxicity, and proved to be highly cell-compatible [117]. Further research has revealed that NG flexible liposomes exhibit superior biocompatibility and therefore play an instrumental role in treating conditions such as in vivo and in vitro bone regeneration [118]. Therefore, the stability, sustained release time, and absorption rate of NG was significantly improved by encapsulating NG in liposomes.

#### 3.4.2. Solid Lipid Nanoparticle 

Nanoparticles are target compounds chemically or physically dispersed on the surface or inside a natural or synthetic carrier material. They form dispersions characterized by particle sizes ranging from 1 to 1000 nm [121]. Compound-loaded nanoparticles improve physicochemical properties by increasing the solubility of poorly soluble substances. Meanwhile, this nanoparticle mitigates target toxicity while retaining the pharmacological activity, therefore safeguarding the target compound against degradation in the gastric environment and enzymes [112]. Moreover, with the increase of specific surface area (the decrease of particle sizes in nanoparticles), the contact efficiency between drugs and cells also increases, suggesting a promising avenue for nanoparticle development potential in pharmaceutical applications [122].

Solid lipid nanoparticles (SLNs) merge the commendable attributes of liposomes and nanoparticles, including cellular affinity, non-toxicity, biodegradability, high stability, acceptable sustained release capabilities, compound-loading capacity, and strong targeting [123]. They constitute a thermodynamically stable dispersion system renowned for its safety. SLNs can adopt varying morphologies besides spherical, including a disk or ellipsoid shapes, with the loaded compound either surface-bound or embedded within the core part [124]. In recent years, nanostructured lipid carriers (NLC, second-generation lipid nanoparticles) have had a small number of liquid lipids incorporated into their structure. This innovative modification effectively mitigates the crystallinity of the lipid core, therefore circumventing compound leakage [125].

Recent research has provided insight into the properties of NG-NLC, revealing them to be uniformly spherical particles with high compound-loading capacity (Figure 4). These carriers have been shown to enhance the effectiveness of anticancer drugs, notably by mitigating chemotherapy side effects and promoting apoptosis. Therefore, Bhia explored SLN carriers loaded with NG and found a notable suppression of anti-apoptotic markers. Concurrently, they observed an up-regulation of Bid mRNA within the pro-apoptotic family, suggesting an increased apoptosis rate. This modulatory action was primarily observed in HT-29 colon cancer cells, where SLN carriers enhanced the overall efficacy of anticancer therapeutics while diminishing the detrimental side effects associated with conventional chemotherapy [119,126]. Furthermore, naringenin SLN enhances solubility and availability. Naringenin SLN was prepared using the emulsification and homogenization method with glyceryl monooleate (GMO) and tocopheryl polyethylene glycol succinate (TPGS) as the co-stabilizer. The results showed that NG-loaded nanoparticles had a long-lasting and sustained release effect for up to 90 h. Significant health benefits were noted in rats exposed to these NG nanoparticles. Their liver enzymes and pro-inflammatory cytokines levels were appreciably reversed, suggesting a potential reduction in liver damage and fibrosis [120]. In conclusion, formulating NG within lipid-based nanocarriers enhances the bioavailability and specificity of its delivery, establishing these nanoparticles as promising vehicles for NG administration. Furthermore, applying NG and drug delivery systems as anticancer agents offers new therapeutic avenues for cancer patients, encouraging the future exploration and application of these novel treatment methods.

#### 3.4.3. Chitosan Nanoparticle

Chitosan (CS), a product of N-deacetylation in chitin, is a natural biological material exhibiting a rich array of properties. After deacetylation, the resultant CS groups are effectively composed of both hydrophilic amino and hydrophobic acetyl groups [127]. Meanwhile, CS possesses some active groups, such as hydroxyl and furan rings (electron-rich), which are electron-rich and could be modified via crosslinking, blending, copolymerization, and ionization [128]. CS and its derivatives exhibit a wide range of properties, such as adsorption, film-forming, fibrillation, permeability, hygroscopicity, biodegradability, biocompatibility, and bacterial inhibition capability, making them promising carrier substances [129].

With inherent positive charges, CS and its nanoparticles resist aggregation due to mutual repulsion [130]. Malathy, et al. prepared NG-loaded chitosan nanoparticles (NCN, particle size 250 nm) with 0.5% CS and 0.12% sodium tripolyphosphate. NCN exhibited promising anti-inflammatory effects. Meanwhile, the coalescence of NG and CS nanoparticles significantly enhanced the inhibitory effect on cancer cells [131]. Specifically, the presence of NCN (100 μM) reduced the viability of human cervical and lung cancer cells by 37% and 30%, respectively. Moreover, a high-dosage NCN treatment has been observed to increase alkaline phosphatase activity, and demonstrates phenomena of collagen and calcium deposition phenomena, signifying its potential in promoting osteoblast differentiation [131].

Additionally, hydrophobic berberine and NG have been encapsulated into CS nanoparticles and incorporated into the chitosan/alginate (CS/ALG) polymer solution to form hydrogel nanocomposites. This complex supports and expedites neurological development and harbors the potential to protect neurons and stimulated cell proliferation [132]. Compared to the control group, the swellable CS/ALG hydrogels of the pure hydrogel group offer a supporting structure for nerve regeneration. Moreover, the CS/ALG hydrogel group shows the capability to promote cell proliferation, and no significant cytotoxicity after 24 inoculating hours is observed. These phenomena suggested that NG hydrogel nanocomposites can significantly improve the functional recovery of damaged nerves [132], positioning them as a new biological material for nerve regeneration.

Liposomes improve the solubility of NGs through vesicle structure. Nanoparticles further improve the physical and chemical properties of the NG complex, while maintaining physiological activity and reducing target toxicity. Both the technology of combining nanoparticles with liposomes and the technology of combining nanoparticles with a biological drug carrier (CS) have made NG stable as a drug in the delivery system, allowing for specific delivery and efficacy at the target sites. Therefore, in future studies, different techniques can be innovatively integrated, and the biological activity of NG with insoluble and low bioavailability can reach its full potential by entering the target site at an effective dose concentration. Thus, the biological activity of NG has been exerted to relieve the corresponding symptoms.

## 4. Conclusions

Naringin has been identified as a compound with considerable potential for clinical application, including treating cardiovascular disease and nervous system disease, modulation of gastrointestinal functions, and facilitation of bone regeneration and repair. Despite these promising applications, the broad adoption of NG in industries such as food, cosmetics, and medicine has been hampered by its inherently low bioavailability, primarily due to its hydrophobic characteristics. Emerging studies have suggested that solubilization methodologies, including structural modification, the preparation of liposomes, inclusion complexes, nanoparticles, and solid dispersions, could enhance the solubility and physiological efficacy of NG. However, the various solubilization methods have limitations. For example, structural modification requires rigorous scrutiny to ensure the final product’s purity and rule out the presence of substances becoming potentially harmful to humans. Furthermore, the encapsulation efficiency and stability of liposomes need to be enhanced, while the tendency of NG to age and crystallize during SD storage must be addressed. In addition, more studies will be needed to explore NG’s absorption and metabolic processes within organisms. A thorough understanding of the metabolism, pharmacokinetics, and safety of NG in vivo remains to be established, thus necessitating further investigation. Therefore, future research trends should focus on optimizing the materials used in preparation methodologies. This could be achieved by applying micro-environment pH regulation technology, bioengineering technology, or combining two different preparation technologies to create innovative solubilization methods. Through these advancements, the bioavailability of NG will be expected to be enhanced, therefore extending its applicability across a broader range of disciplines. Meanwhile, exploring NG’s synergistic effects with other compounds and studying their interactive dynamics represents promising research avenues. In-depth exploration of these areas could unlock new opportunities and applications for naringin, further enriching its contribution to food, cosmetics, and medicine.

## Data Availability

The data that support the findings of this study are available from the corresponding author upon reasonable request.
